# Datura Poisoning in Trinidad: A Case Report

**DOI:** 10.7759/cureus.29829

**Published:** 2022-10-02

**Authors:** Camille Jaggernauth, Dominic Dalip, Peng Ewe, Shiva Jaggernauth

**Affiliations:** 1 Emergency Medicine, Southern Medical Clinic, San Fernando, TTO; 2 Internal Medicine, Leicester Royal Infirmary, Leicester, GBR; 3 Seattle Science Foundation, Seattle, USA; 4 Internal Medicine, Southern Medical Clinic, San Fernando, TTO; 5 Anaesthesiology, Southern Medical Clinic, San Fernando, TTO; 6 Respiratory Medicine, Apley Medical Clinic, San Fernando, TTO; 7 Pulmonary Medicine, Southern Medical Clinic, San Fernando, TTO

**Keywords:** anticholinergic overdose, toxicity, anticholinergic poisoning, stimulant, datura poisoning

## Abstract

Datura, a wild-growing annual plant, common in the American Southwest and the Caribbean, has many uses, including medicinal or pharmaceutical, ornamental, religious, and social. In the Caribbean, this white trumpet-shaped flower has been used for many cultural aspects and has also been found to be used as a potent psychological stimulant. Despite its many purposes, its inappropriate misuse can result in mild-to-severe toxicity, leading to severe anticholinergic effects and even death in some cases. The purpose of this report is to highlight the toxic effects of this plant when misused and the subsequent management as it relates to the spectrum of anticholinergic poisoning, a common complication of drug overdose. We describe a case of datura poisoning presenting in Trinidad, West Indies, which was not described previously in the literature.

## Introduction

Datura belongs to the *Solonaceae* family within the plant kingdom and is comprised of 9 to 12 species, which includes *Datura stramonium* and *Datura metel* [1]. These two species are the most common in the South American region [2]. The species can be differentiated based on their physical characteristics. They are known by several other names: Jimson weed, Locoweed, Angel’s Trumpet, Thorn apple [2]. Datura flowers are used worldwide for its host of medicinal properties ranging from anti-nociceptive, anti-inflammatory, anti-spasmodic, to central nervous system (CNS) stimulant effects. It is used as a respiratory decongestant, in the treatment of dental infections/toothache, skin infections, and alopecia, and to relieve abdominal colicky pain [3-5]. Culturally, it is used as part of ritual offerings called "jhalls" in the form of a blended mixture or as the whole flower itself for “Shivaratri,” an internationally celebrated annual Hindu festival. Over a billion devotees throughout the diaspora worldwide observe this festival in honor of Lord Shiva.

Of more concern, parts of the plant are used by adolescents in some parts of the world as a form of a recreational substance due to its hallucinogenic and euphoric effects [6]. This plant can cause toxicity to the cardiovascular, respiratory, and neurological systems. Upon review of the literature, there were no published case reports on Datura poisoning in Trinidad and Tobago. We describe a case of Datura poisoning presenting at Southern Medical Clinic (SMC), Trinidad, West Indies.

## Case presentation

A 75-year-old male was brought to the Emergency Department at Southern Medical Clinic after being found unconscious and incontinent on the bathroom floor by his relatives for an unknown duration of time after being last seen 16 hours prior in his normal state of health. His medical history includes hypertension, hyperlipidemia, and sarcoidosis. On admission, his vital signs were the following: blood pressure of 161/71 mm Hg, heart rate of 108 beats per minute, respiratory rate of 26 breaths per minute, temperature of 36°C, oxygen saturation of 97% on room air, and random blood glucose of 161 mg/dL.

His neurological examination revealed a Glasgow Coma Score (GCS) that fluctuated between 8 and 9 (E 2-3, V1, M5) with bilateral “blown” pupils. He was agitated, and his skin was flushed. Tone was increased globally, power was 4/5 in all muscle groups, and Babinski reflexes were positive bilaterally. There were no focal deficits or signs of meningeal irritation, and the rest of his examination was normal with no evidence of trauma.

Blood investigations including full blood count, metabolic profiles, and arterial blood gas were within normal range, while bedside urine analysis revealed 3+ blood and urine toxicology negative (Table 1). Electrocardiogram showed a sinus tachycardia with normal PR and QTc intervals, while radiological investigations including CT brain revealed no abnormalities. Further questioning of his relatives revealed that the patient may have ingested an unknown quantity of Datura tea after participating in Shivaratri festivities. The working diagnosis at this point was acute anticholinergic poisoning secondary to ingestion of Datura tea. The patient was then transferred to the intensive care unit (ICU) for further monitoring, observation, and supportive management.

**Table 1:** Laboratory investigations for day 1 and day 3 WBC, white blood cell count; HB, hemoglobin; PLT, platelets; BUN, blood urea nitrogen; Cr, creatinine; Na, sodium; K, potassium; Cl, chloride; Ca, calcium; Alb, albumin; Mg, magnesium; P, phosphorus; CK, creatine kinase; ALT, alanine transaminase; AST, aspartate aminotransferase; PTT, partial thromboplastin time; PT, prothrombin time; INR, international normalized ratio

Blood chemistry			
Parameters	Day 1	Day 3	Reference range
WBC, 10^3^/uL	11.3	8.7	4.0–10.5
HBG, g/dL	11.6	10.8	11.5–16.5
PLT, 10^3^/uL	229	150	150 - 400
BUN, mg/dL	32	24	6 - 20
Cr, mg/dL	1.5	1.0	0.5–1.2
Na, mmol/L	139	141	135–145
K, mmol/L	4.9	4.2	3.5–5.1
Cl, mmol/L	106	104	98 - 107
Ca, mg/dL	8.4	8.1	8.4–10.2
Alb, g/dL	3.3	3.1	3.5–5.0
Mg, mg/dL	1.8	1.7	1.60–2.60
Phosphorus, mg/dL	2.9	2.6	2.3–4.7
CK, U/L	13,880	2345	29.0–168.0
CK-MB, ng/mL	47	4	0.0–3.1
MYOGLOBIN, ng/mL	6076.9	392	0.0–106.0
TROPONIN, ng/mL	0.138	0.032	0.000–0.015
AST, U/L	316	113	5.0–34.0
ALT, U/L	57	38	0.0–55.0
PTT, seconds	31.2		20.0–30.0
PT, seconds	12.3		11.0–13.0
INR	1.0		<1.2

In the ICU, neostigmine 5 mg x two doses administered intravenously in 5-minute intervals (no contraindication) resulted in a brief improvement in GCS from 8 to 11 and reduction of pupillary size to 3 mm bilaterally. Although the patient was able to nod his head in response to questioning and obeyed commands initially, he became agitated again shortly afterward, requiring sedation with 5 mg of midazolam.

Due to the agitation and decreased GCS, the patient was intubated to protect his airway. Intubation was achieved with remifentanil via target-controlled infusion (TCI), propofol 80 mg, and rocuronium 30 mg. He was ventilated on synchronized intermittent ventilation (SIMV) mode with continuous sedation using remifentanil TCI and midazolam. Neostigmine infusion at 0.5 mg/hr was commenced with dose adjustment based on hourly pupillary assessment. As the patient also had rhabdomyolysis with creatine kinase (CK) level of 13,880 U/L and myoglobin level of 6,076.6 ng/mL, forced diuresis was established with 0.9% sodium chloride fluids at 4 L/24 hours and intermittent dosing of furosemide to maintain a urine output >75 mL/hour to prevent renal impairment. A nasogastric tube was inserted where a large volume of gastric contents was aspirated, and 100 grams of activated charcoal was administered later.

Intermittent sedation breaks were undertaken to assess neurological status, and the patient was able to be extubated after 24 hours as his GCS improved to 15/15 (E4, M6, V5) with no further agitation. He was able to obey commands, and his pupils were 2.5 mm bilaterally reactive to light. The neostigmine infusion was discontinued after 36 hours. Considering that the ECG and echocardiogram were normal, the mildly elevated troponin level was attributed to rhabdomyolysis. His CK and myoglobin levels continued to decrease to baseline on repeat blood sampling. His repeat urine toxicology screen was positive for cannabis metabolites, although the initial admission screen was negative. It was unclear as to why the initial urine toxicology screen did not show positive cannabis.

Post-extubation, the patient revealed that he blended 5 pods of Datura seeds, approximating 400-500 seeds, a quarter ounce of marijuana, 250 mL of honey, and 100 mL of milk, of which he ingested 200-250 mL. At 72 hours post-admission, the patient’s vital signs were as follows: blood pressure of 135/71 mm Hg, heart rate of 74 beats per minute, respiratory rate of 20 breaths per minute, and oxygen saturation of 99% on room air. The patient's neurological status was stable, GCS was 15/15, and he was discharged with advice and outpatient follow-up.

## Discussion

The Datura flower contains 64 alkaloids, but of particular importance are the belladonna alkaloids (tropane alkaloids) that comprise a methylated nitrogen atom along with the anticholinergic drugs - atropine, scopolamine, and hyosine [2,3,7]. These alkaloids act as competitive antagonists to peripheral and muscarinic acetylcholine receptors, leading to a state of general paralysis of the parasympathetic innervated organs, resulting in the manifestation of the classical anticholinergic syndrome [3,8]. Acute psychosis occurs due to the tertiary amines inhibiting the CNS, likely due to the antagonism of post-synaptic M1 receptors [8].

**Figure 1:**
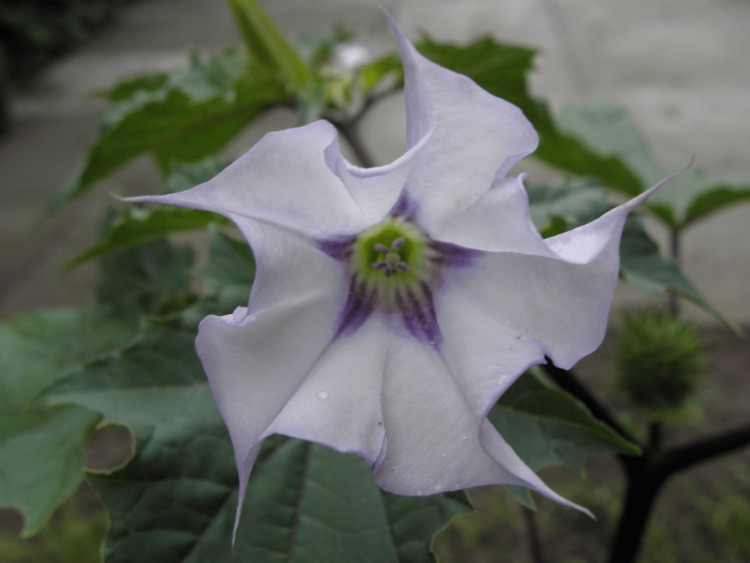
Datura stramonium (funneled-shaped and white-colored)

**Figure 2:**
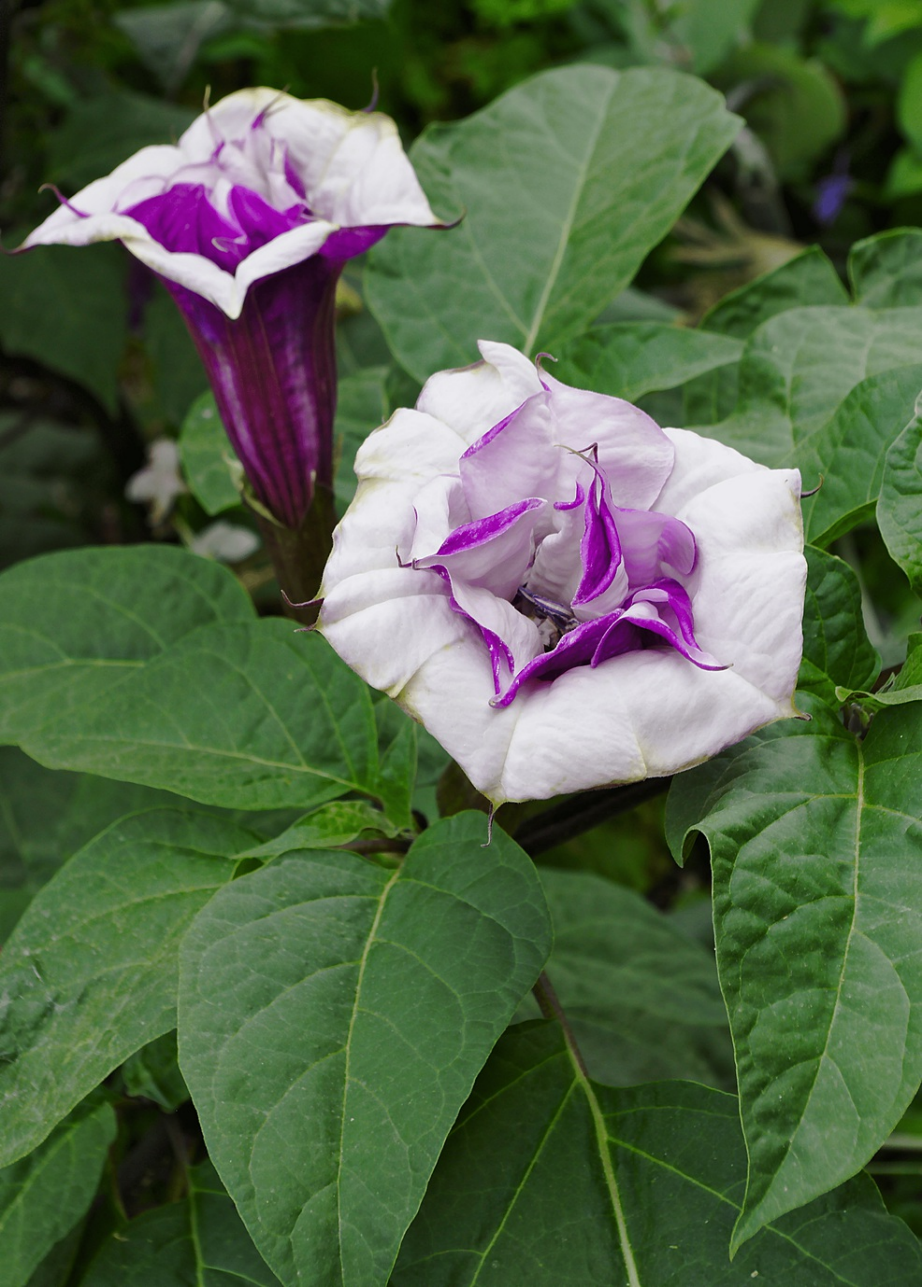
Datura metel (funneled-shaped and purple-colored)

Inappropriate consumption of any part of the plant results in anticholinergic poisoning but the extent of toxicity is highly variable and unpredictable depending on the part of the plant ingested [6]. Datura has pointed leaves and large funnel-shaped white- or purple-colored flowers (Figures 1, 2). Its fruit capsule or pod is spherical with soft spines and contains up to 200 small dark brown or black reniform seeds that resemble chilly seeds [3]. The highest concentration of toxins is found in the seeds, in particular the ripe seeds [9], where boiling or drying destroys their poisonous properties; 50 to 100 seeds approximate to 3-6 mg of atropine. Toxicity occurs when ingested, smoked, or absorbed topically via mucous membranes. The fatal dose comprises of 50 to 100 Datura seeds, which equates to 10-100 mg of atropine and 2-4 mg of scopolamine; however, studies have shown recovery with greater than 500 mg of atropine equivalent [3,6].

Datura poisoning resembles the classic picture of atropine intoxication and is represented by numerous symptoms and signs that can be subdivided into peripheral and central effect [5,6,10]:

· Dry skin and mucosa, flushing, hyperpyrexia (dry skin may help distinguish anticholinergic toxidromes from sympathomimetic toxidromes)

· Mydriasis, neurological impairment with ataxia, short-term memory, disorientation, confusion, hallucination, psychosis, delirium, and agitation, as well as seizures and coma in severe cases

· Sinus tachycardia

· Decreased intestinal motility, ileus, urinary retention

· Rhabdomyolysis and fulminant hepatitis in some severe cases

· Respiratory and cardiovascular collapse in extreme cases but rare

Toxicity occurs within 30 to 60 minutes following ingestion and can persist up to 24 to 48 hours owing to delayed gastric emptying [1,6]. Management of datura poisoning is focused on monitoring, observation, and supportive care.

When anticholinergic toxicity is strongly suspected due to ingestion of Datura or any plant containing tropane alkaloids, gastric decontamination with activated charcoal is performed at a dose of 1 g/kg and can be repeated within 12 hours [10]. Gastric lavage is also critical to prevent the reabsorption of toxins via enterohepatic circulation [1,8]. Gastric decontamination is of particular importance since reduced gastrointestinal motility leads to prolonged absorption and delayed peaks, and thus prolonged effects of the anticholinergic toxidrome.

Intubation and mechanical ventilation are necessary as a means of a secured airway in preventing aspiration and providing respiratory support [1-6]. Continuous sedation with intermittent breaks to allow neurological assessment to carry out early extubation thereby, minimizing the complications associated with intubation and mechanical ventilation [6]. Patients may be sedated with benzodiazepines to control agitation and psychosis [10].

Aggressive fluid administration aids in the treatment of rhabdomyolysis that results from direct toxicity of alkaloids and secondary to myoglobinuria [1,5,6]. The aim is to prevent renal failure. Diuretics may also be necessary when assessing the patient’s input/output. Urinary retention is also part of the anticholinergic toxidrome, exacerbating the delirium in some cases. Hemodialysis will not aid in the removal of Datura alkaloids but may be required if acute renal failure or severe refractory hyperkalemia is present [3].

Treatment of hyperpyrexia with cooling measures and paracetamol contributes to the resolution of the tachycardia as well as fluid therapy [6]. It is imperative that the patient be assessed for evidence of cardiotoxicity such as QT or QRS prolongation as a necessity to general management and risk assessment for the use of cholinesterase inhibitors [8].

Physostigmine, a diagnostic and therapeutic agent, is a tertiary ammonium compound that reverses both central and peripheral anticholinergic effects as it is a reversible acetylcholinesterase inhibitor. The ideal antidote in the management of anticholinergic syndrome is a selective M1 receptor antagonist. The recommended initial dose is 0.5 to 2 mg intravenously over 5 minutes with repeated doses if necessary [1,10,11]. This antidote is recommended in the presence of severe agitation and psychosis, tachycardia or dysrhythmias with cardiac instability, seizures, and coma. Of note, there are some relative contraindications to the use of physostigmine and they include bradydysrhythmias, intraventricular and atrioventricular blocks, bronchospasm, intestinal obstruction, bladder obstruction, and peripheral vascular disease [10]. However, the clinical background of same cases may require the use of physostigmine and thus, in these instances, it can be used in a reduced dose of 0.5 mg [6]. Physostigmine is sometimes administered cautiously to distinguish functional psychosis from anticholinergic delirium.

In our case, physostigmine was not available, but neostigmine was readily available as part of medication used routinely to reverse neuromuscular blockade in the operating theater. The patient’s clinical condition only improved briefly with bolus doses of neostigmine before deteriorating again; thus, an intravenous infusion was warranted. The writers of this case acknowledge that physostigmine is the most reported antidote in numerous studies, even though neostigmine is similar. The only difference between both drugs is that the tertiary amine structure of physostigmine allows it to penetrate the blood-brain barrier, thereby exerting central cholinergic effects, whereas neostigmine, being a quaternary ammonium compound, is unable to penetrate the CNS [3,8,12]. However, neostigmine, by acting peripherally, was able to reverse the peripheral anticholinergic effects, while sedation was used to counter the central psychotic effects and agitation from the poisoning.

By day 2, the patient’s neurological status improved dramatically where he was obeying commands, was oriented, and had multiple bowel movements, indicating the reversal of the anticholinergic effects on bowel. The patient's GCS was 15/15 at this time. He was able to be extubated by 24 hours owing to the supportive measures that were undertaken and the use of neostigmine to control the peripheral effects induced by the anticholinergic toxidrome. Studies have shown that neostigmine is used as medical therapy in reversing colonic ileus coupled with gastric lavage [12].

## Conclusions

Anticholinergic toxicity due to plant ingestion should be considered as a differential diagnosis when other objective findings are lacking in patients who present with acute delirium, agitation, and altered mental status. Accidental poisoning may occur from mistaken identity of the seeds, such as children who chew on the seeds out of curiosity or therapeutic misadventures. However, a diagnostic problem seems to be common because of the presentation with a variety of clinical symptoms, incomplete medical histories, misguided efforts by patients themselves, relatives, or friends, and, lastly, delayed or untimely laboratory/chemical analysis.

Fatalities due to Datura poisoning are rare, but physicians and individuals should be aware of its anticholinergic effects as it is readily available in Trinidad and is routinely used for various religious undertakings. Worldwide, Hindu devotees who annually celebrate this festival of Shivaratri are at risk of such toxicity as it is used in part of the ritualistic offerings and celebrations. Educating doctors, Hindu priests, and the Hindu population of its potentially life-threatening effects should be actively pursued. Careful consideration should be undertaken before its use due to its lethal properties.

## References

[1] Korkmaz MF, Bostancı M, Onur H, Cagan E (2018). Datura stramonium poisoning: a case report and review of the literature. Eur Res J.

[2] Trancă SD, Szabo R, Cociş M (2017). Acute poisoning due to ingestion of Datura stramonium - a case report. Rom J Anaesth Intensive Care.

[3] Rasila Devi M, Bawari M, Paul SB, Sharma GD (2011). Neurotoxic and medicinal properties of datura stramonium. Assam Univ J Sci Technol.

[4] Pillay VV, Sasidharan A (2019). Oleander and datura poisoning: an update. Indian J Crit Care Med.

[5] O’Connor SE (2010). Alkaloids. Chemistry, Molecular Sciences and Chemical Engineering.

[6] Debnath T, Chakraverty R (2017). Newer insights into the psychoactive and pharmacological properties of Datura stramonium Linn. Glob J Add & Rehab Med.

[7] Kohnen-Johannsen KL, Kayser O (2019). Tropane alkaloids: chemistry, pharmacology, biosynthesis and production. Molecules.

[8] Soni P, Siddiqui AA, Dwivedi J, Soni V (2012). Pharmacological properties of Datura stramonium L. as a potential medicinal tree: an overview. Asian Pac J Trop Biomed.

[9] Ogunmoyole T, Adeyeye RI, Olatilu BO, Akande OA, Agunbiade OJ (2019). Multiple organ toxicity of Datura stramonium seed extracts. Toxicol Rep.

[10] Dawson AH, Buckley NA (2016). Pharmacological management of anticholinergic delirium - theory, evidence and practice. Br J Clin Pharmacol.

[11] Nickalls RW, Nickalls EA (1988). The first use of physostigmine in the treatment of atropine poisoning. A translation of Kleinwachter's paper entitled 'Observations on the effect of Calabar bean extract as an antidote to atropine poisoning'. Anaesthesia.

[12] Isbister GK, Oakley P, Whyte I, Dawson A (2001). Treatment of anticholinergic-induced ileus with neostigmine. Ann Emerg Med.

